# Antimicrobial and anti-biofilm activity of *Polygonum chinense* L.aqueous extract against *Staphylococcus aureus*

**DOI:** 10.1038/s41598-022-26399-1

**Published:** 2022-12-20

**Authors:** Jianye Zeng, Dandan Chen, Chunli Lv, Kening Qin, Qin Zhou, Na Pu, Shanshan Song, Xiaomin Wang

**Affiliations:** 1 School of Preclinical Medicine of Zunyi Medical University, Zunyi Medical University, Zunyi, 563000 Guizhou People’s Republic of China; 2 National Clinical Research Center for Infectious Diseases, Shenzhen, 518112 Guangdong People’s Republic of China

**Keywords:** Drug discovery, Medical research, Drug development, Bacterial infection

## Abstract

*Polygonum chinense* Linn. (*Polygonum chinense* L.) is one of the main raw materials of Chinese patent medicines such as Guangdong herbal tea. The increasing antibiotic resistance of *S. aureus* and the biofilm poses a serious health threat to humans, and there is an urgent need to provide new antimicrobial agents. As a traditional Chinese medicine, the antibacterial effect of *Polygonum chinense* L. has been reported, but the antibacterial mechanism of *Polygonum chinense* L.aqueous extract and its effect on biofilm have not been studied in great detail, which hinders its application as an effective antibacterial agent. In this study, the mechanism of action of *Polygonum chinense* L.aqueous extract on *Staphylococcus aureus* (*S. aureus*) and its biofilm was mainly evaluated by morphological observation, flow cytometry and laser confocal experiments. Our findings demonstrate that *Polygonum chinense* L.aqueous extract has a significant bacteriostatic effect on *S. aureus*. The result of growth curve exhibits that *Polygonum chinense* L.aqueous extract presents a significant inhibitory effect against *S. aureus*. Transmission electron microscopy (TEM) and scanning electron microscopy (SEM) reveals that *Polygonum chinense* L.aqueous extract exerts a potent destruction of the cell wall of *S. aureus* and a significant inhibitory effect on the formation of *S. aureus* biofilm. In addition, flow cytometry showed the ability of *Polygonum chinense* L.aqueous extract to promote apoptosis by disrupting cell membranes of *S. aureus*. Notably, confocal laser scanning microscopy (CLSM) images illustrated the ability of *Polygonum chinense* L.aqueous to inhibit the formation of *S. aureus* biofilms in a dose-dependent manner. These results suggested that *Polygonum chinense* L.aqueous is a promising alternative antibacterial and anti-biofilm agent for combating infections caused by planktonic and biofilm cells of *S. aureus*.

## Introduction

Infectious diseases are the second leading cause of death worldwide^1^. *S. aureus*, a gram-positive bacterium, can cause variety of infections ranging from minor skin and soft tissue infections such as impetigo, folliculitis, and cutaneous abscesses to life-threatening diseases such as sepsis, infective endocarditis or toxic shock syndrome respiratory, tract and bloodstream infections^2,3^. Besides, *S. aureus* can produce a variety of toxins including enterotoxins related to food poisoning, toxic shock syndrome toxin, hemolysin, leukocidin and exfoliative toxins, which generate various diseases ranging from skin infections to systemic life-threatening diseases^4,5^. During the past decade, there has been an increasing awareness that *S. aureus* biofilms are a major cause for concern in multiple infections^6^. As reported by the National Institutes of Health (NIH), over 60% of all bacterial and 80% of chronic bacterial infections are associated with biofilm formation^7^. Meanwhile, infections caused by biofilm formation of *S. aureus* are hard to treat^8^. In American, approximately 93.5% of chronic leg ulcers are infected with *S. aureus*, and bacterial biofilms are the leading cause of treatment failure in chronic wounds^9^. Biofilm formation during chronicity is one of the staphylococcal strategies resulting in the failure of antibacterial therapy, which increases the cost and delays the patient’s recovery^10^. The ability of *S. aureus* to form biofilms and persisters is a major cause of recalcitrant infections that are difficult to treat^11^. Given the rapid emergence of drug-resistant *S. aureus* but a lack of antibiotic-development pipeline, alternative strategies are urgently needed to combat antibiotic-resistant *S. aureus*^12^.

In recent years, many studies have explored that plant extracts, such as phenols and flavonoids, showed good antibacterial activities^13^. *Polygonum chinense* Linn. (*Polygonum chinense* L.), a perennial herb, belongs to the family Polygonaceae and is a common medicinal plant in China, India, Japan, and Southeast Asian countries^14,15^. *Polygonum chinense* L. has been revealed that the plant contains terpenoids, alkaloids, flavonoids, tannins, steroids and glycosides^15^. Flavonoids are the main component category, and quercitrin is the main component of flavonoids^16^. Its methanol (MeOH) extract showed antimicrobial, antioxidant and cytotoxic activities in vitro^15^. The aim of this study was to investigate the anti-biofilm and antibacterial activity of its aqueous extracts against *S. aureus*.

## Results

### MIC and MBIC of *Polygonum chinense* L.aqueous extract against *S. aureus*

*Polygonum chinense* L.aqueous extract against *S. aureus* displayed minimum inhibitory concentration (MIC) of 4 mg/mL and minimum biofilm inhibitory concentration (MBIC) of 16 mg/mL.

### Effect on bacterial growth

**Figure 1:**
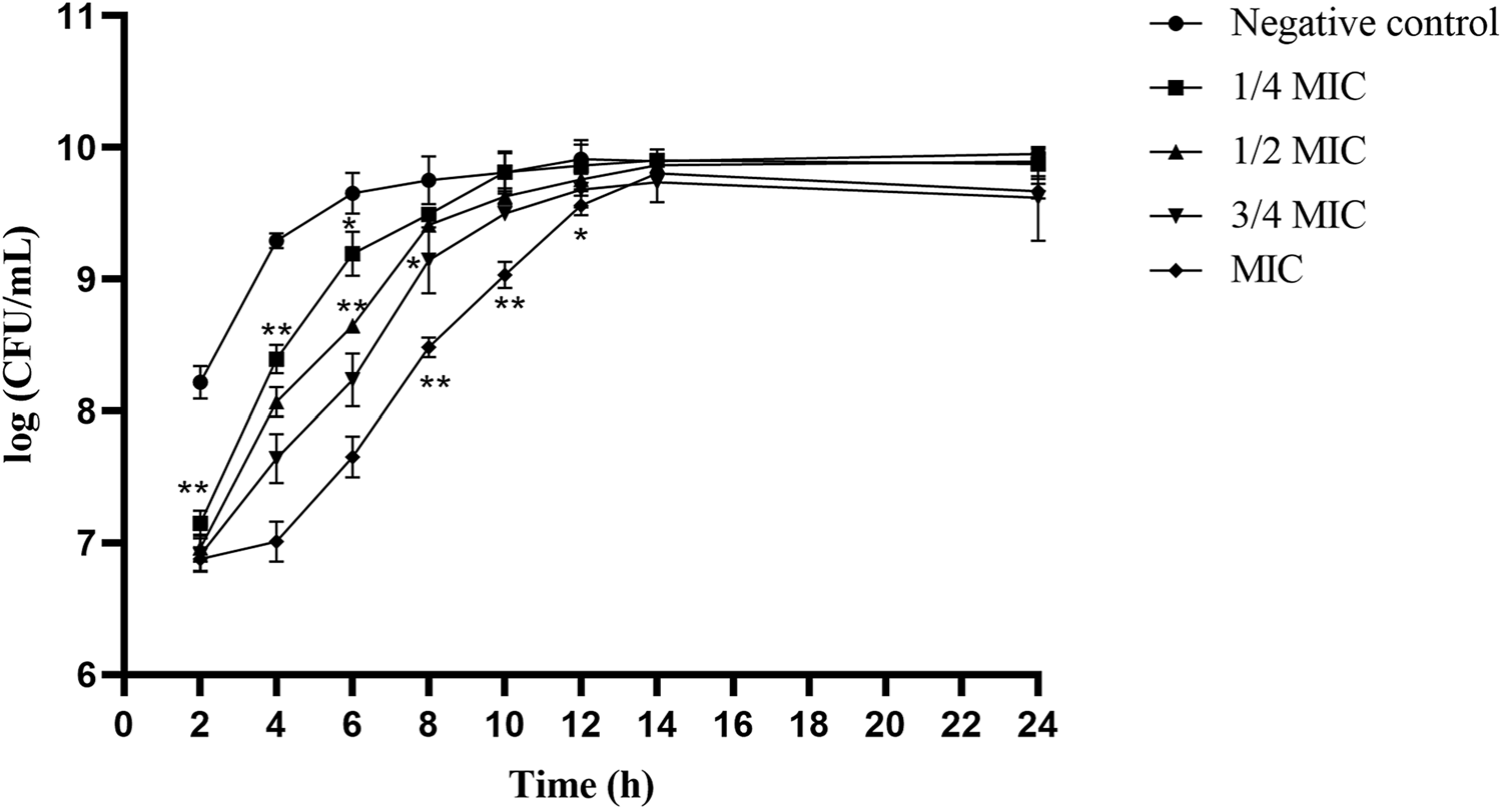
The effect of *Polygonum chinense* L.aqueous extract on the growth curve of *S. aureus*.

Following determination of the MIC of *Polygonum chinense* L.aqueous extract against *S. aureus*, the bacterial growth curve was plotted at different time points to compare the growth situation with or without *Polygonum chinense* L.aqueous extract exposure (Fig. 1). *Polygonum chinense* L.aqueous extract showed remarkable zone of inhibitions against *S. aureus* in a concentration-dependent manner. Significant inhibition of *S. aureus* growth within 12 h when treated with *Polygonum chinense* L.aqueous extract at MIC concentration.

### Effect of *Polygonum chinense* L.aqueous extract on cell morphology

**Figure 2:**
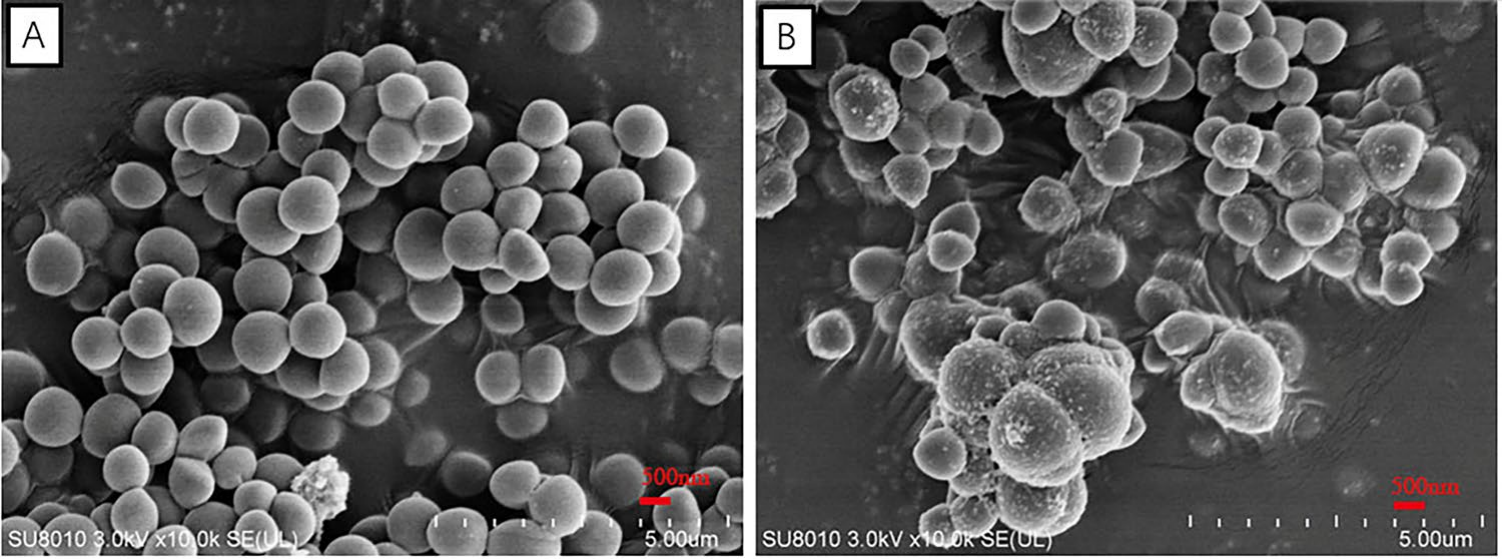
Morphological structure of *S. aureus* observed by SEM. (**A**) Untreated; (**B**) *Polygonum chinense* L.aqueous extract treatment 20 h.

Figure 2 presented the morphological changes of both the treated and untreated *S. aureus*. These images illustrated the disruptive effect of *Polygonum chinense* L.aqueous extract on *S. aureus* visually. Untreated *S. aureus* cells displayed the normal morphological characteristics. They showed smooth, round, and spherical surfaces with clear boundaries. Besides, the cells were arranged in clusters of grapes under SEM (Fig. 2A). After treatment for 20 h with *Polygonum chinense* L.aqueous extract, there were obvious vesicular on the surface, loose and irregular arrangement in cells. In addition, the cell volume became larger after treatment. In summary, large amounts of malformed cells were observed in the microphotograph of *S. aureus* (Fig. 2B). This result demonstrated that *Polygonum chinense* L.aqueous extract caused strong damage of the bacterial cell wall to exert antimicrobial effects.

**Figure 3:**
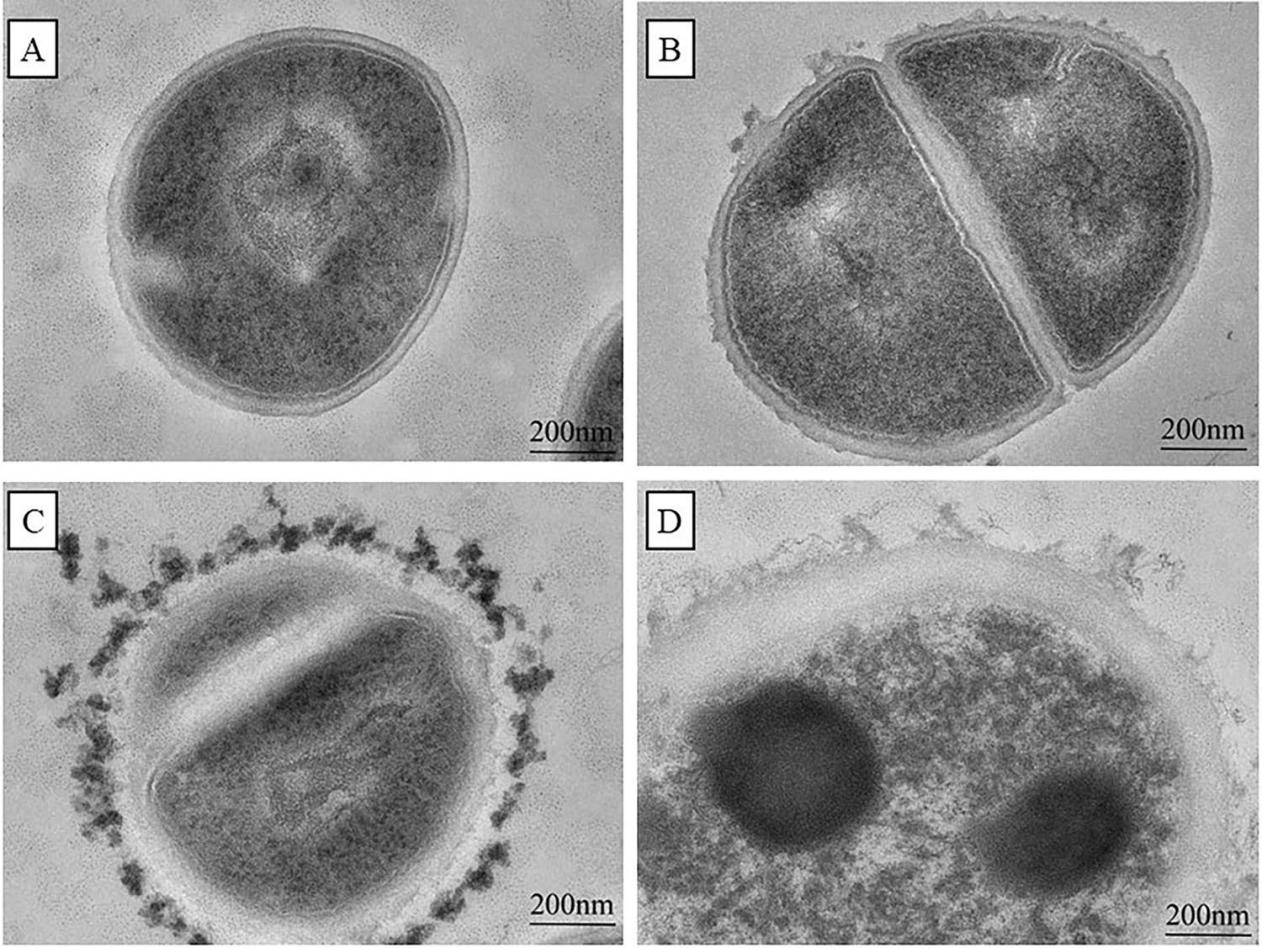
Morphological structure of *S. aureus* observed by *Polygonum chinense* L.aqueous extract. (**A**) Untreated; (**B**) *Polygonum chinense* L.aqueous extract treatment 2 h; (**C** and **D**) *Polygonum chinense* L.aqueous extract treatment 6 h.

The changes of ultrastructure of *S. aureus* were examined by using TEM (Fig. 3). In control group (Fig. 3A), the cells were surrounded by the cell membrane with compact surface, showing a well-defined cell membrane, a smooth cell wall and an uniform cytoplasm region, and without release of intracellular components. After 2 h incubation with *Polygonum chinense* L.aqueous extract, cell wall and membrane were dissolved and the shape of cells became irregular; In addition, the uniformity of the cytoplasm region was disturbed and unequal cell division could be observed (Fig. 3B). After treatment for 6 h, cells were seriously damaged (Fig. 3C, D). There was a loss of cell integrity, and the cytoplasmic contents were leaking out of the cells. The serious damage of cell wall and membrane result in the cell boundary became blurred and the extracellular solutes existed. Besides, the bacteria autolysis occurred in the treated cells (Fig. 3D).

### Effect on cell apoptosis

**Figure 4:**
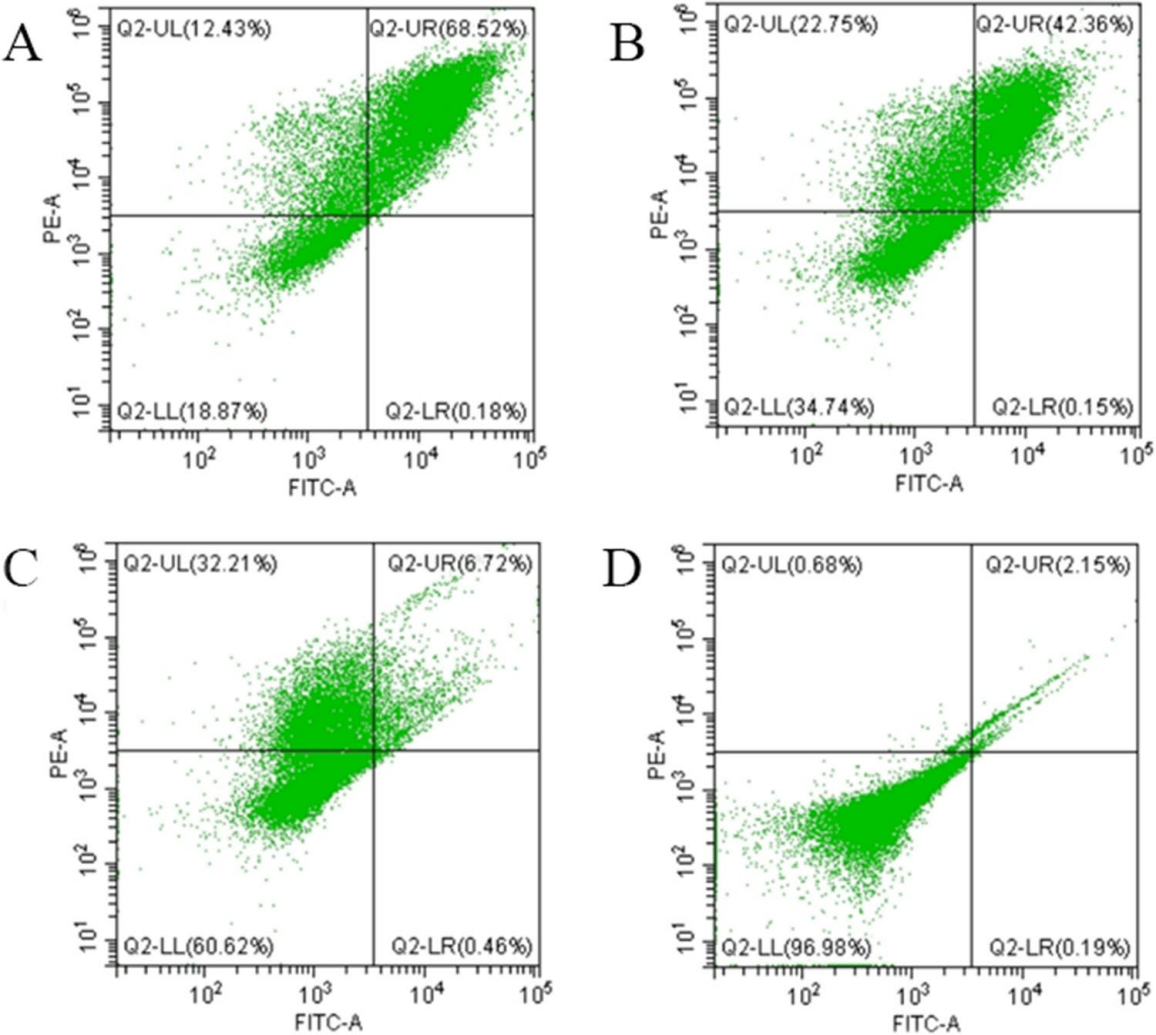
Cell apoptosis of *S. aureus* treated with different concentrations of *Polygonum chinense* L.aqueous extracts. (**A**) 2 × MIC; (**B**) 1 × MIC; (**C**) 1/2 × MIC; (**D**) Control.

To explore the effect of *Polygonum chinense* L.aqueous extract on the cell membrane, flow cytometry was applied and the results are presented in Fig. 4A–D. As PI is a membrane impermeable dye, it cannot enter viable cells with intact membrane, which is shown in Q2-LL district. According to the Fig. 4, the proportion of live cells was 18.87%, 34.74%, and 60.62% after treating with *Polygonum chinense* L.aqueous extract at level of 2 × MIC, 1 × MIC, and 1/2 × MIC, respectively, which was much less than 96.98% in the control group. Additionally, the results further confirmed that the antibacterial activity of *Polygonum chinense* L.aqueous extract against *S. aureus* depends on its dose.

### Characterization of the biofilm microstructure

**Figure 5:**
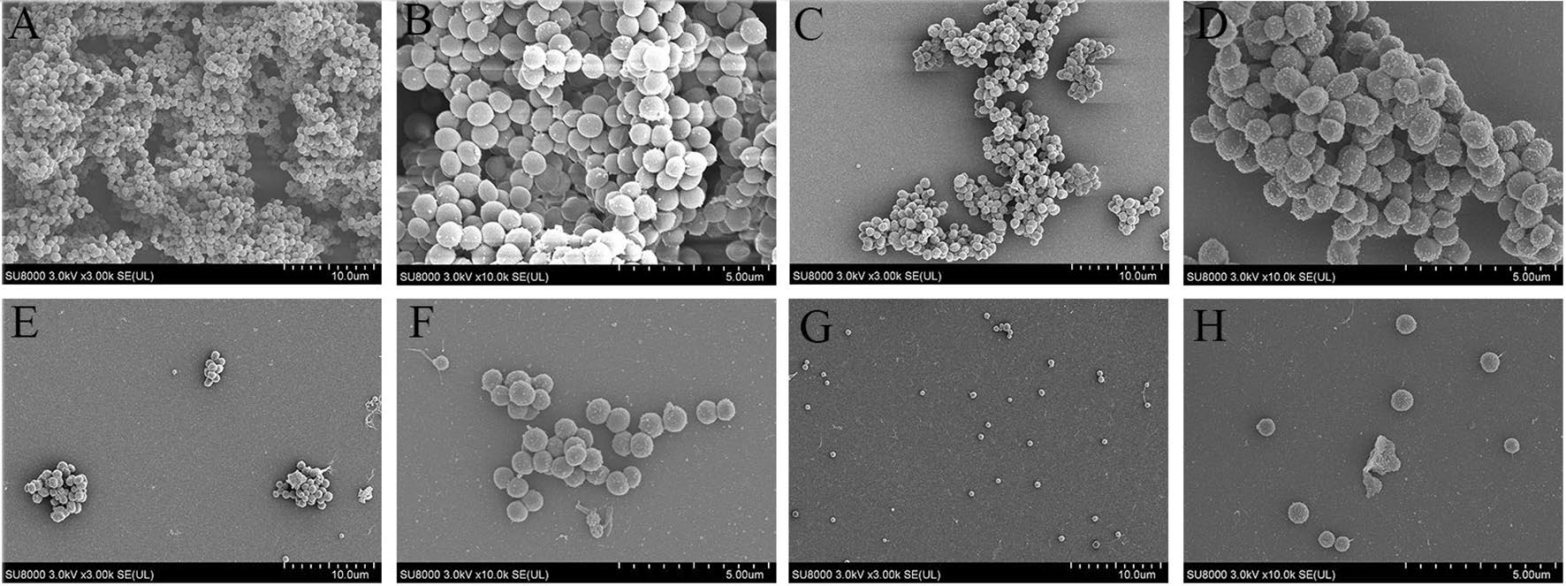
Biofilm microstructure conditions observed by SEM. (**A**) and (**B**) Control; (**C**) and (**D**) 1 mg/mL; (**E**) and (**F**) 2 mg/mL; (**G**) and (**H**) 4 mg/mL.

The *S. aureus* biofilms grown with and without *Polygonum chinense* L.aqueous extract were examined by SEM (Fig. 5). The results revealed a significantly decreased biofilm growth in treated groups compared to untreated group. *S. aureus* formed a thick biofilm made of aggregates and the cells connected to each other to organize 3-dimensional structure manner. However, the number of cells and the extracellular matrix of the biofilm were significantly reduced and the cells scattered in treated samples. In addition, the total number of bacteria obviously decreased as the concentration of *Polygonum chinense* L.aqueous extract increased.

### CLSM observation of biofilm formation

**Figure 6:**
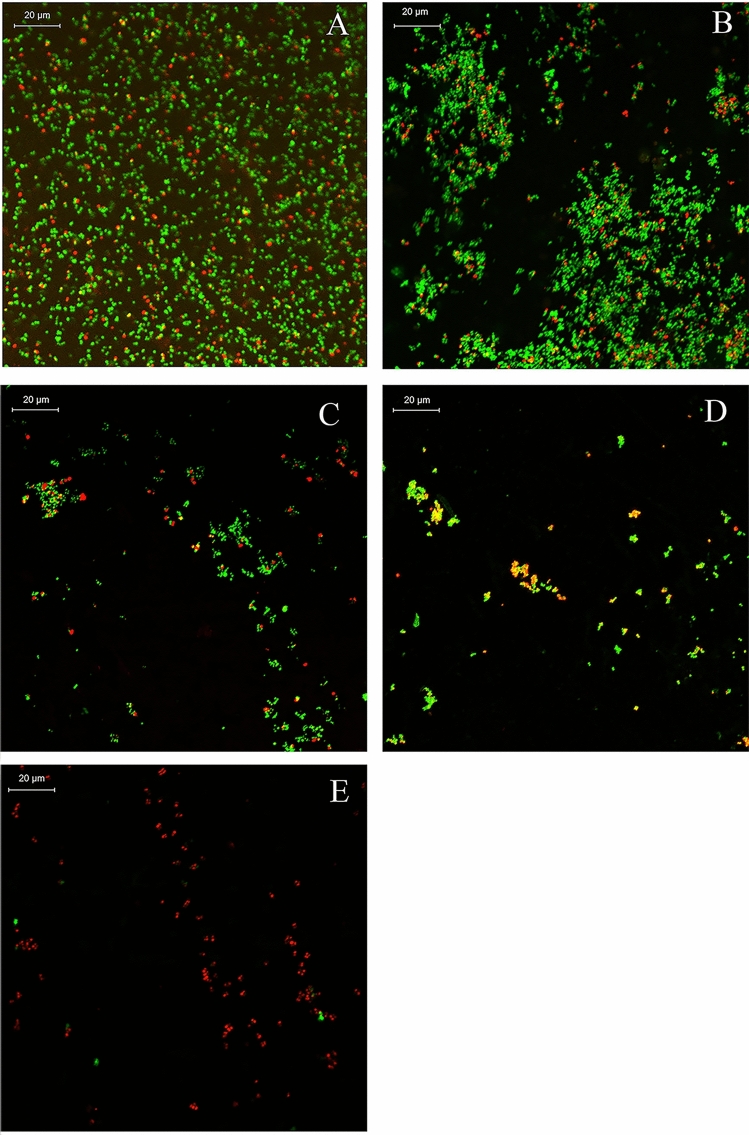
CLSM images of *S. aureus* biofilms. (**A**) Control; (**B**) 0.25 mg/mL; (**C**) 0.5 mg/mL; (**D**): 1 mg/mL; (**E**) 2 mg/mL. Live bacteria stains green, dead bacteria stains red.

The anti-biofilm activity of *Polygonum chinense* L.aqueous extract was further confirmed through CLSM (Fig. 6). CLSM observations and digital image analysis showed that the surface of the control group was rough and the fluorescence signal of living bacteria was strong (Fig. 6). When treated with *Polygonum chinense* L.aqueous extract, the biofilm biomass of *S. aureus* obviously decreased. As the concentration of *Polygonum chinense* L.aqueous extract increased, the extracellular polysaccharides and bacterial cells in the biofilm decreased. The CLSM biofilm formation results were similar to SEM.

### The effects of *Polygonum chinense* L.aqueous extract on *S. aureus* biofilm formation

**Figure 7:**
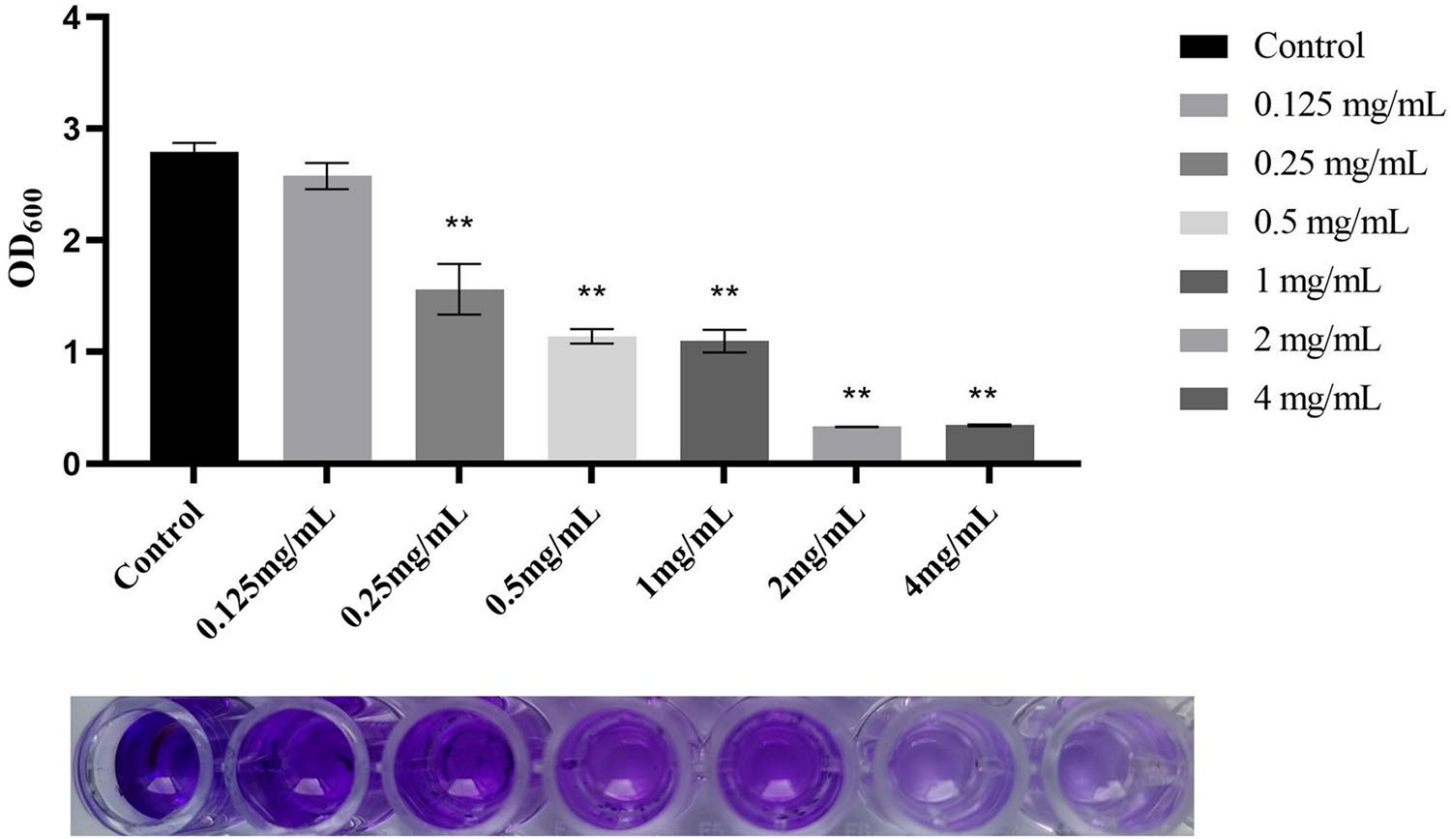
The effects of *Polygonum chinense* L.aqueous extract on *S. aureus* biofilm formation. *P < 0.05; **P < 0.01.

Inhibition of biofilm formation by *Polygonum chinense* L.aqueous extract was compared with the control by the crystal violet assay for each biofilm. The results demonstrated that *Polygonum chinense* L.aqueous extract had an inhibitory effect on the biofilm formation in a dose-dependent manner (Fig. 7). Besides, a significant difference was observed between the treated groups and the control group when the concentration of *Polygonum chinense* L.aqueous extract reached 0.25 mg/mL. The above observations of CV assay were consistent with CLSM and SEM, thus further confirm the anti-biofilm activity of *Polygonum chinense* L.aqueous extract.

### Effect of *Polygonum chinense* L.aqueous extract on eDNA release from *S. aureus*

**Figure 8:**
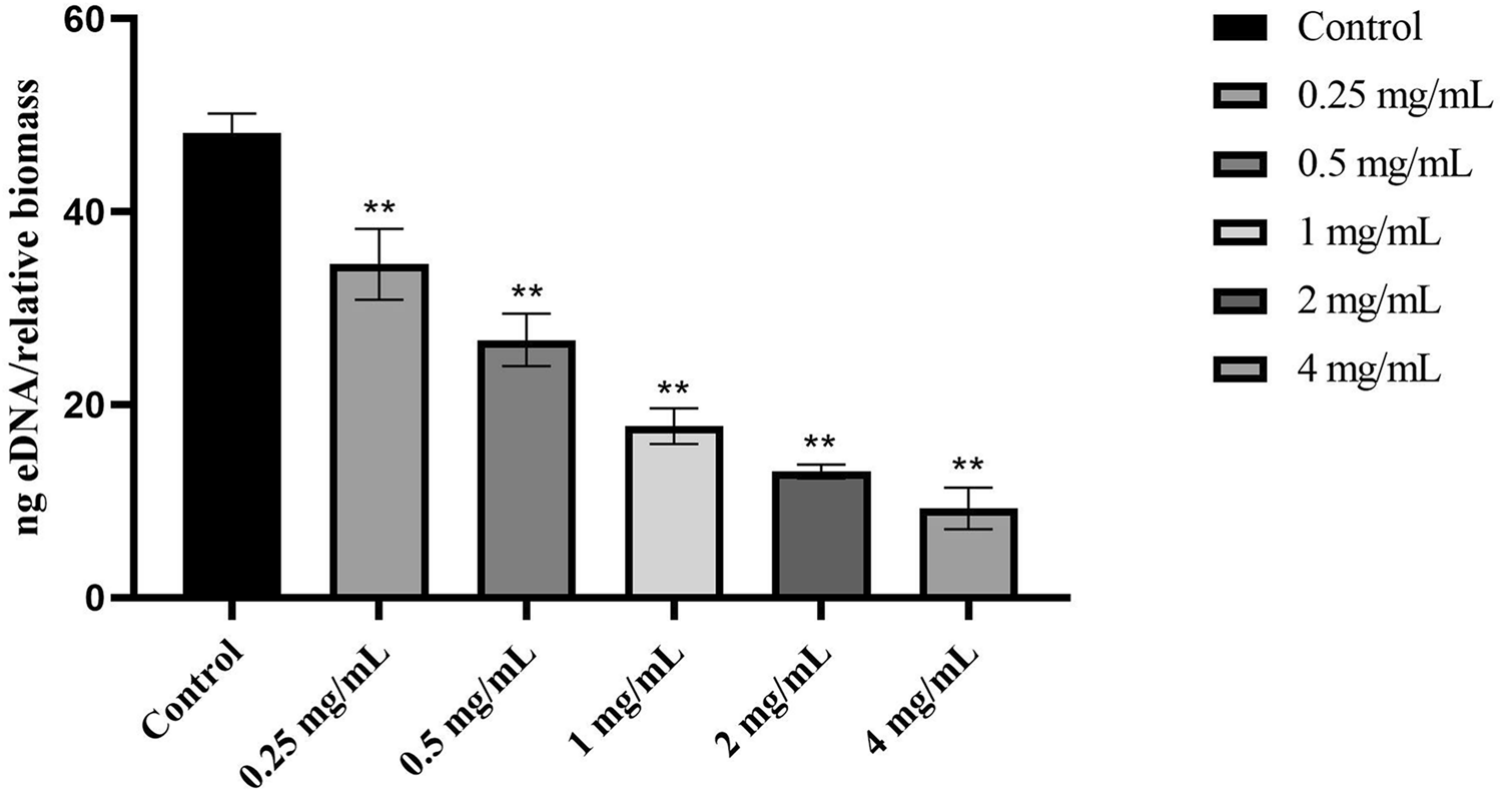
Effect of *Polygonum chinense* L.aqueous extract on the eDNA release in the *S. aureus* biofilm. *P < 0.05; **P < 0.01.

To better understand the effect of *Polygonum chinense* L.aqueous extract on biofilm formation, the quantity of eDNA released from the biofilms was assessed in the absence and presence of *Polygonum chinense* L.aqueous extract. As shown in Fig. 8, compared with the untreated group, the amount of eDNA was significantly reduced when the concentration of *Polygonum chinense* L.aqueous extract reached 1 mg/mL in a dose-dependent manner.

### Biofilm eradication assay

**Figure 9:**
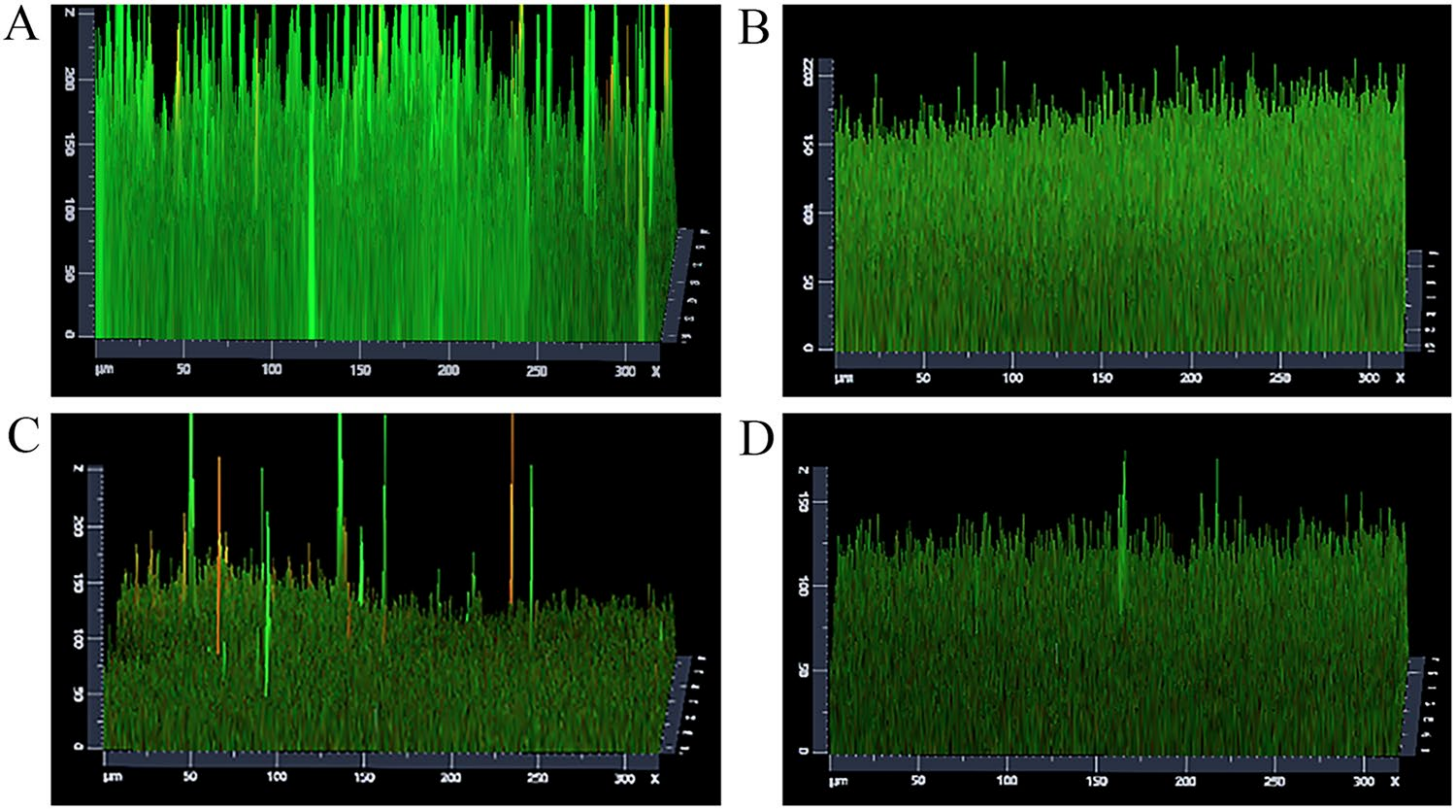
Effect of *Polygonum chinense* L.aqueous extract on the *S. aureus* biofilm. (**A**) Control; (**B**) 8 mg/mL; (**C**) 12 mg/mL; (**D**) 16 mg/mL.

The CLSM observation (Fig. 9) indicates that the amount of *S. aureus* biofilm decreased to varying degrees after treatment with 8 mg/mL, 12 mg/mL, 16 mg/mL of *Polygonum chinense* L.aqueous extract compared with the control group, and 12 mg/mL, 16 mg/mL of *Polygonum chinense* L.aqueous extract treatment groups decreased more significantly than the 8 mg/mL *Polygonum chinense* L.aqueous extract treatment group.

## Discussion

With the emergence of multidrug-resistant *S. aureus*, the need for more effective treatment of biofilm-associated infections has become imperative^17^. Therefore, it is very important to investigate antimicrobial agents to treat the infection of *S. aureus*. Natural extracts from plants have many unique advantages for the treatment of *S. aureus* biofilm infections^18^. In the study, *Polygonum chinense* L.aqueous extract was evaluated for its antibacterial and anti-biofilm activity. The results indicated that *Polygonum chinense* L.aqueous extract had antibacterial activity against *S.aureus*, and its MIC was 4 mg/mL. In the bacterial growth curve, all the groups treated with *Polygonum chinense* L.aqueous showed obvious growth inhibition relative to control. Therefore, their antibacterial mechanism was further studied. SEM and TEM are universally used for the study of antimicrobial mechanisms. Results from SEM and TEM micrographs showed *Polygonum chinense* L.aqueous extract disrupted the structural integrity of the cell wall. In addition, TEM results revealed that the *S. aureus* treated with *Polygonum chinense* L.aqueous extract showed unequal fission, cell autolysis, the shrink of cytoplasm, plasmolysis and leakage of cell contents. Gallic acid, an ellagic acid compound in *Polygonum chinense* L., can interact with many targets in the bacterial cell wall, disrupting the structure of the cell wall and making the cell wall synthesis process inefficient^16,19^. Therefore, we speculate that the severe disruption of *S.aureus* cell walls may be caused by gallic acid in *Polygonum chinense* L.aqueous extract. Therefore, the mechanism of action of *Polygonum chinense* L.aqueous on the membrane of *S. aureus* was further explored. The essential function of cell wall and membrane is to serve as a selective permeability barrier to separate the cell from its external environment^13,20^. In this study, flow cytometric analysis was used to detect the integrity and permeability of cell membrane. The PI staining data confirmed tha *Polygonum chinense* L.aqueous induced membrane destruction. The cell membrane is the main site where flavonoids act on Gram-positive bacteria^21^. So the destruction of cell membrane may be the result of the action of flavonoids in *Polygonum chinense* L.aqueous extract. In addition, *Polygonum chinense* L.aqueous has an inhibitory effect on the development of biofilms in a concentration-dependent manner by performing SEM, CLSM and CV assay. The results showed that *Polygonum chinense* L.aqueous extract inhibited the formation of biofilm. Studies have shown that quercetin, a flavonoid, prevents bacterial adhesion and inhibits the quorum-sensing pathway^22^. Therefore, quercetin in *Polygonum chinense* L.aqueous extract may play a role in this process. To clarify possible mechanism of the decreased biomass of biofilm in the presence of *Polygonum chinense* L.aqueous, the influence of *Polygonum chinense* L.aqueous on the eDNA in *S. aureus* biofilm was investigated. As shown in Fig. 8, the eDNA content in *S. aureus* biofilm of treatment with *Polygonum chinense* L.aqueous extract significantly decreased in a dose-dependent manner. It has been reported that phenolic compounds can mimic the quorum sensing signal, thereby reducing the release of eDNA^23^. In addition, DNase can also degrade the released eDNA. Whether the reduction of eDNA in EPS is caused by the production of DNase to degrade the released eDNA or by inhibiting the release of eDNA remains a question that requires more research. All data taken together suggest that *Polygonum chinense* L.aqueous extract has the potential to be applied as an antibacterial agent against *S. aureus* and to inhibit *S. aureus* biofilm formation and reduce *S. aureus* biofilm biomass in vitro.

## Conclusions

The intractable clinical infection caused by biofilms poses a serious threat to public health, among which *S. aureus* biofilms account for the majority^24,25^. Here we report the antimicrobial and anti-biofilm activity of *Polygonum chinense* L.aqueous extract against *S. aureus*. Our findings show that *Polygonum chinense* L.aqueous extract not only killed *S. aureus* cells through multiple mechanisms but also inhibited the formation of *S. aureus* biofilms. In future work, we will further investigate its antibacterial mechanism and potential applications in food and pharmaceutical industries.

## Statistical analyses

Statistical analysis was carried out using SPSS Statistics 18.0 software. Differences between groups were significant at the level of *P* < 0.05 in this study.

## Methods

### Microorganisms and chemicals

*S. aureus* ATCC-29213 was provided by the Laboratory of Microbiology and Immunology, Zunyi Medical University. It was grown on Trypticase (Tryptic) Soy Broth/Agar (TSB/TSA) at 37 °C and maintained at 4 °C for future use. All the chemicals used in this study were of analytical grade. Three triplicates were performed for every experiment.

### Preparation of *Polygonum chinense* L.aqueous extract

*Polygonum chinense* L.aqueous extract was prepared as previously described with some modifications^17^. Fresh *Polygonum chinense* L. was collected from Zunyi City, Guizhou Province. Plant species were identified by Researcher Li-Hong Wu, Institute of Chinese Materia Medica, Shanghai University of Traditional Chinese Medicine. A voucher specimen of the plant (HTMHE023) was deposited at the Herbarium of the college of Traditional Chinese Medicine, Shanghai University of Traditional Chinese Medicine, China. The impurities such as dust, scum, and other particles were removed from the gathered *Polygonum chinense* L. by washing it with tap water, and then distilled water thrice. After powdered fresh *Polygonum chinense* L. was dried to a constant weight in 60 °C, it was powdered and passed through a 40-mesh sieve for further use (Tianchuang Powder Technology Co., Ltd., Changsha, China). 100 g of powder were accurately weighed and soaked overnight in deionized water (2000 mL; yield) at room temperature and then heated until boiling for 30 min. Filters were used in the extraction process to obtain the filtrate. Subsequently, the filtrate were collected and the medicinal residues were subsequently added into distilled water again, then the above extraction was repeated. The filtrate was concentrated under reduced pressure on a rotary evaporator, and the concentrated solution was freeze-dried into powder at low temperature. When used, it was diluted with deionized water, which was *Polygonum chinense* L.aqueous extract.

### Minimum inhibitory concentration and minimum biofilm inhibitory concentrations

Detection of MIC is the minimum quality concentration that significantly inhibits the growth of *S. aureus*.

MIC assays were performed as described previously with some modifications^26^. *Polygonum chinense* L.aqueous extract was added to the nutrient broth medium, giving final concentrations of 1–1000 mg/mL. The control was the *S. aureus* culture containing aseptic distilled water without *Polygonum chinense* L.aqueous extract. And then 100 μL bacterial suspension was collected and serially diluted in phosphate-buffered saline (PBS), and 100 μL of which was plated onto the nutrient agar plates and incubated for 24 h in a 37 °C incubator.

To confirm minimum biofilm inhibitory concentration (MBIC), a previously described method was used with some modifications^27^. 200 μL of bacterial culture (~ 10^6^ CFU/mL) was aliquoted into a 96-well plate. The plate was then incubated for 24 h at 37 °C to form mature biofilm formation. After the medium was discarded, the cells were washed three times with PBS to remove extracellular bacteria. And then, 200 μL of TSB medium containing *Polygonum chinense* L.aqueous extract in serial doubling dilutions was added to each well. TSB medium without *Polygonum chinense* L.aqueous extract was used as a negative control, and the plate was incubated at 37 °C for 24 h. After the medium was discarded, the cells were washed once with PBS and resuspended in 250 µL of PBS. Subsequently, each sample was sonicated for 30 min in an ultrasonic bath (25 °C, 250 W, 50 Hz) in order to resuspend the biofilm cells thoroughly. 10 μL was removed from each well and spot plated onto the TSA plate and incubated for 24 h in a 37 °C incubator. MBIC was determined as the lowest concentration whose visible colony numbers is equal to or less than the control’s colonies on the agar plate.

### Bacterial growth curve

The bacterial growth curve was performed as previously described, with some modifications^13,28^. *S. aureus* was grown overnight at 37 °C with shaking at 200 rpm in TSB medium. 1 mL of the suspension was removed and adjusted to 2 × 10^8^ CFU/mL as seeds. Then, 1 mL of seed solution was inoculated into centrifuge tubes containing 9 mL of sterile TSB and *Polygonum chinense* L.aqueous extract (1/4 × MIC, 1/2 × MIC, 3/4 × MIC and MIC). *S. aureus* cultures without *Polygonum chinense* L*.*aqueous *extract* served as a control. Then, the medium was incubated at 37 °C and 200 rpm. Serial tenfold dilutions (100 μL in triplicate) were seeded on TSA at 2, 4, 6, 8, 10, 12, 14 and 24 h. The colony-forming units (CFUs) were counted after 18–24 h incubation at 37 °C.

### SEM and TEM

The SEM assays were performed as previously described, with some modifications^29,30^. SEM was used to observe the morphological changes of bacteria after exposure to *Polygonum chinense* L.aqueous extract. Briefly, cells were treated with final 2 mg/mL *Polygonum chinense* L.aqueous extract at 37 °C for 20 h. The absence of *Polygonum chinense* L.aqueous extract was used as a control. Then bacterial cells were centrifuged at 4500 rpm for 15 min to pellet down and washed three times with PBS. After centrifugation, the bacterial precipitate was fixed with 2.5% glutaraldehyde at 4 °C for 24 h. After three washes with PBS, samples were post-fixed for 2 h in 1% OsO4 reagent. A pretreatment step consisting of a series of dehydrating steps in ethanol was performed before they were infiltrated with epoxy resin. Gold–palladium coated dehydrated samples were sputtered by Hitachi Model E-1010 ion sputter for 5 min and analyzed by Hitachi Model SU-8010 SEMs. An experiment was conducted without *Polygonum chinense* L.aqueous extract as a control.

The TEM assays were performed as previously described, with some modifications^31,32^. TEM was used to observe the ultrastructural features of cells. Briefly, the cell suspensions (~ 10^9^ CFU/mL) were incubated with *Polygonum chinense* L.aqueous extract at 4 mg/mL for 2 h and 6 h incubation respectively. No *Polygonum chinense* L.aqueous extract treatment served as the control group. Cell pretreatment used to prepare TEM assays was the same as for SEM. After dehydration, the cells were embedded in epoxy resin and the resin was stored at 55 °C for 48 h to allow resin polymerization and then cut into thin sections (approximate 70 nm). After three washes with PBS, samples were post-fixed for 2 h in 1% OsO_4_ reagent. Then the embedded samples were sliced with a thickness of 50–70 nm. The sections were prepared on copper grids and stained with 2% uranyl acetate and lead citrate. A HT7700 (Hitachi, Japan) was used to observed the changes of cells.

### Evaluation of cell apoptosis

The experiment was performed as described previously with some modifications^33^. Apoptosis was monitored using Annexin V-FITC/PI Apoptosis Detection Kit. *Polygonum chinense* L.aqueous extract was added to the bacterial suspension (~ 2 × 10^7^ CFU/mL) at final concentrations of 1/2 × MIC, 1 × MIC and 2 × MIC, respectively. The absence of *Polygonum chinense* L.aqueous extract was used as a control. Cells were incubated at 200 rpm for 6 h at 37 ℃. Then, the samples were washed with sterile water. Next, Annexin V-FITC was added to the bottom of the well and incubated for 15 min in the dark. The stained cells were analyzed by flow cytometry using a BD FACSCalibur cell analyzer (BD Biosciences). Data was analyzed using CytExpert software (Beckman Coulter).

### Analysis of biofilms using SEM

The assay was performed according to the previously reported method with some modifications^34^. SEM was used to directly observe the effect of *Polygonum chinense* L.aqueous extract on biofilm formation. In brief, Logarithmic phase cells of *S. aureus* (~ 10^6^ CFU/mL) was cultivated overnight at 37 °C for 24 h in a 6-well microtiter plate with glass slides and supplemented with *Polygonum chinense* L.aqueous extract at the designated concentrations of 1–4 mg/mL. The absence of *Polygonum chinense* L.aqueous extract was used as the control. Subsequently, the wells were washed three times with PBS and fixed with 2.5% glutaraldehyde at 4 °C for 12 h. Then biofilms were observed by SEM.

### CLSM analysis

The assay was performed according to the previously reported method with some modifications^35,36^. Cell viability was determined using Dead Viability Cytotoxicity Assay Kit (UE Landy). NucGreen stains all cells, but EthD-III only stains cells with damaged membrane. 1 mL logarithmic phase cells of *S. aureus* suspensions (~ 10^6^ CFU/mL) were seeded into 6-well plates with cell climbing slice. The assays were performed in the presence of *Polygonum chinense* L.aqueous extract (0.25, 0.5, 1 and 2 mg/mL). The absence of *Polygonum chinense* L.aqueous extract was used as a control. After 24 h incubation, unattached cells were removed by rinsing the cell climbing slices with PBS. The biofilms were incubated in the NucGreen / EthD-III staining mixture for 15 min in the dark. Then the slides were washed three times with PBS and observed under CLSM (LSM 800, Carl Zeiss, Jena, Germany).

### CV assay

The assay was performed according to the previously reported method with some modifications^37,38^. 200 μL logarithmic phase cells of *S. aureus* suspensions (~ 10^6^ CFU/mL), contained *Polygonum chinense* L.aqueous extract with various concentrations of 0.125, 0.25, 0.5, 1, 2 and 4 mg/mL in TSB respectively. The absence of *Polygonum chinense* L.aqueous extract was used as a control. After 24 h of incubation at 37 °C, the culture medium was removed, and PBS was used to wash the wells three times. After the plates were dried, 200 μL of methanol was added to the wells for fixation (15 min). Then the Biofilms were stained with 1% crystal violet (200 µL) dye for 15 min at room temperature, washed and dried naturally. After that, 200 μL of absolute ethanol was added to dissolve the biofilm and incubated at 37 °C for 1 h. Absorbance at 600 nm (OD_600_) was recorded.

### eDNA quantification

The assay was performed according to the previously reported method with some modifications^39,40^. Logarithmic phase cells of *S. aureus* (~ 10^6^ CFU/mL) was grown on 6-well plate with each well containing 2 mL of TSB with and without *Polygonum chinense* L.aqueous extract (0.25, 0.5, 1, 2 and 4 mg/mL) and incubated at 37 °C for 24 h. TSB without treatment was used as a control. Then, biofilms chilled at 4 °C for 1 h after 1 mL of TEN buffer was added to each well. Next, TSB was removed, and the wells were washed carefully with 0.85% normal saline to remove planktonic cells. 700 μL of TE buffer was added to remove adhered biofilm cells. Cells were transferred to 1.5 mL microcentrifuge tubes and pelleted by centrifugation at 16,000 rpm for 5 min at 4 °C. The eDNA in the supernatant was extracted with phenol–chloroform-isoamyl alcohol (25:24:1), precipitated with absolute alcohol, and resuspended in 50 μL of TE buffer. eDNA was quantified by using a Take3 spectrophotometry system on a Synergy HI microplate reader (BioTek, USA).

### Effect against mature biofilms

Biofilm eradication assay was performed as described previously with some modifications^41,42^. The cell climbing slices were placed in 6-well microplates, into which suspensions of exponentially grown bacteria (10^6^ CFU/mL, 2 mL/well, in TSB) were added. After culturing at 37 °C for 24 h to form biofilms, the spent culture medium was removed and the biofilms were washed three times with sterile PBS to remove planktonic cells and spent culture medium. Then the biofilms were exposed to different concentrations of *Polygonum chinense* L.aqueous extract (8 mg/mL, 12 mg/mL and 16 mg/mL) for 12 h. The control group was added with the same amount of PBS solution. The biofilms were then stained with 50 μg/mL fluorescein isothiocyanate-concanavalin A (FITC-Con A) (Sigma, USA), which binds extracellular polysaccharide (representing biofilms) and 5 μg/mL propidium iodide (PI) (dead cells) (BD Biosciences, USA) for 20 min. The biofilms were observed by CSLM.

### Ethics approval and consent to participate

All plant experiments described in this study complied with relevant institutional, national, and international guidelines and legislation.

## Data Availability

The data used or analyzed during the present study are available from the corresponding author on reasonable request.
